# Gonadotropin-releasing hormone and cognition

**DOI:** 10.1210/endocr/bqaf033

**Published:** 2025-02-27

**Authors:** Loïc Kacimi, Vincent Prevot

**Affiliations:** Univ. Lille, Inserm, CHU Lille, Laboratory of Development and Plasticity of the Neuroendocrine Brain, Lille Neuroscience & Cognition, FHU 1000 days for health, EGID, DistALZ, UMR_S112, Lille, France

**Keywords:** minipuberty, GnRH pulsatility, GnRH receptor, brain, menopause, aging

## Abstract

Gonadotropin-releasing hormone (GnRH) is traditionally recognized as the central regulator of reproduction through its pulsatile secretion, which governs the hypothalamic-pituitary-gonadal (HPG) axis. However, recent evidence has highlighted its broader role in brain development and function, including in cognitive and higher intellectual processes. GnRH production follows distinct phases, from its early activation during minipuberty – the first postnatal activation of GnRH neurons during the infantile period, its reactivation and stabilization starting at puberty, and its eventual decline with age and the loss of gonadal steroid feedback. This evolution depends on the establishment, maturation and activation of GnRH neurons, a complex process regulated by the cellular and molecular environment of these neurons, including multiple neuronal and glial types as well as a minipubertal “switch” in gene expression, the perturbation of which may have long-term or delayed consequences for both reproductive and cognitive function. The cognitive role of GnRH may be related to its recently revealed involvement in maintaining myelination and synaptic plasticity, while disruptions in its finely tuned rhythmic secretion, either age-related or pathological, are associated with cognitive decline and neurodegenerative disorders. Restoring physiological GnRH levels and pulsatility can reverse age-related cognitive decline and improve sensory functions even in adulthood, suggesting a mobilization of the “cognitive reserve” in both animal models and human patients. This review highlights recent advances in our understanding of the GnRH system and the therapeutic potential of pulsatile GnRH therapy to mitigate age-related cognitive decline and neurodegenerative processes.

## Introduction

Gonadotropin-releasing hormone (GnRH) is a decapeptide neurohormone produced and released by a small group of neurons, around 2,000 out of the hundred billion neurons in the human brain and approximately 800 neurons in mice (1,2), that ensures the survival of the species by acting as the master regulator of reproductive function (3,4). Neuroendocrine GnRH neurons project to the median eminence (ME) of the hypothalamus and release GnRH into the pituitary portal circulation in a distinct pattern essential for its function, characterized by low-level basal secretion interspersed with rhythmic pulses in both males and females as well as a preovulatory surge in females (5–7). Through the portal circulation, GnRH reaches the anterior pituitary gland, where it induces the secretion of the gonadotropins, luteinizing hormone (LH) and follicle-stimulating hormone (FSH), from gonadotrope cells by binding to its cognate receptor, GnRH-R (8). In mammals, including humans, the frequency of GnRH pulses is finely regulated, and each GnRH pulse released by the brain triggers a corresponding pulse of LH in the bloodstream, where it can be measured as a proxy for GnRH (9–13). LH and FSH then reach the gonads and stimulate gonadal growth, gametogenesis and the synthesis of sex steroids (primarily testosterone in males, and estrogens and progesterone in females) (14,15). These gonadal steroids in turn provide either positive or negative feedback to various levels of the hypothalamic-pituitary-gonadal (HPG) axis (13,16,17), finetuning its function, including the pattern of GnRH secretion.

Over the past 15 years, transgenic rodent models expressing β-galactosidase (18), green fluorescent protein (GFP) (19–21), CRE recombinase (22–24), as well as viral vectors have significantly advanced our ability to study the molecular, cellular, and functional properties of GnRH neurons and the neuroglial network controlling the production and release of the neuropeptide, as well as those of its GnRH-R expressing targets (25,26). Recently, the use of these unique genetic animal models and tools has led to the unexpected discovery that GnRH neurons and GnRH also play a significant role in the development and maintenance of cognitive, sensory and behavioral processes, including various higher-order tasks, as well as normal and pathological brain aging (27–29). Emerging evidence suggests that every aspect of the development, connectivity, function and maintenance of GnRH neurons and their complex cellular and molecular environment may play a role not only in correct reproductive function but in the regulation of these novel roles.

This review provides a concise overview of recent advances in our understanding of the role of GnRH in cognitive function.

### Neuroanatomy and physiology of GnRH neurons

**Figure 1:**
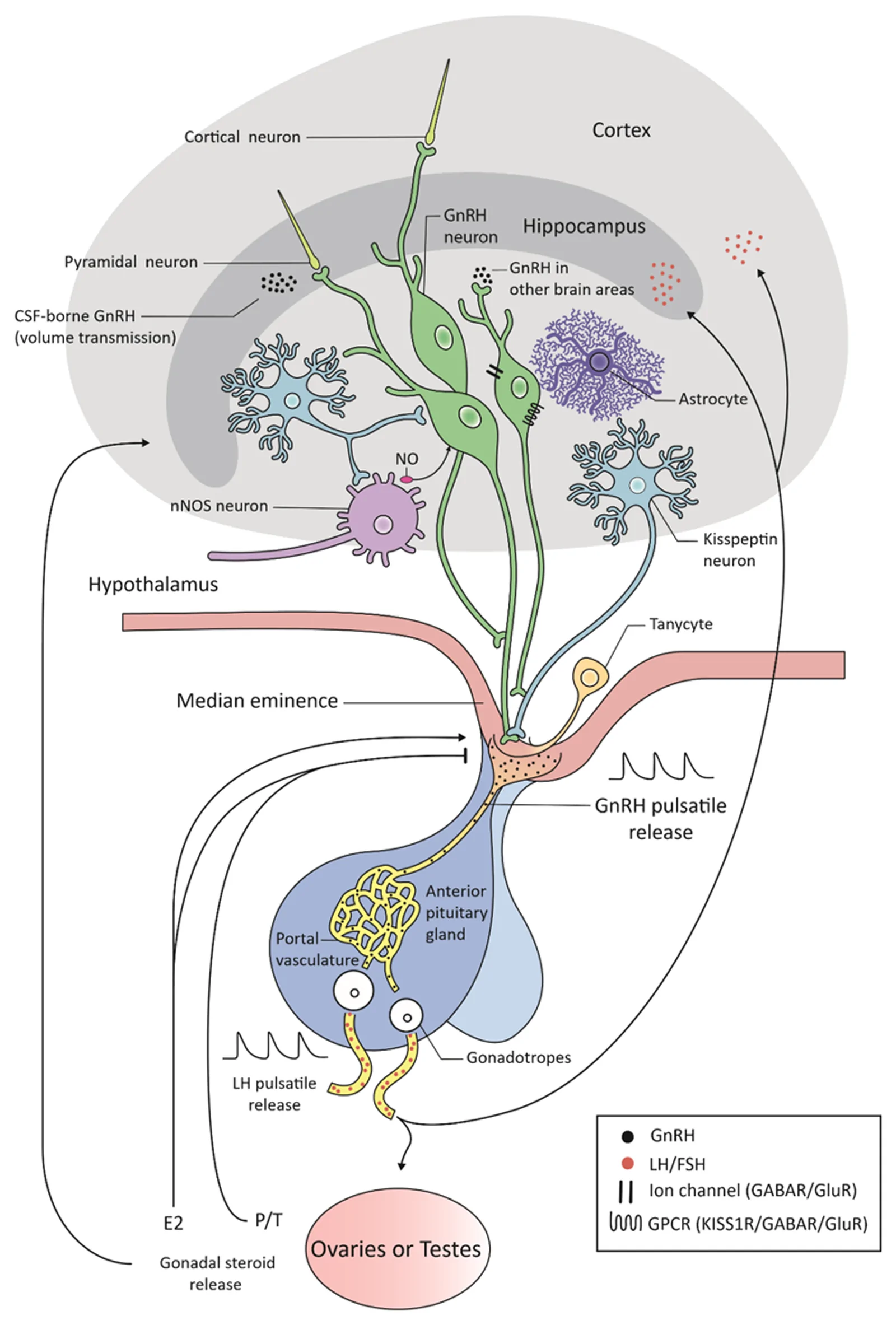
The hypothalamic–pituitary–gonadal axis. Neuroendocrine neurons producing gonadotropin-releasing hormone (GnRH) are distributed principally in the preoptic region of the hypothalamus and project to the median eminence (ME), where GnRH is released in a pulsatile manner into pituitary portal blood vessels for delivery to the anterior pituitary gland. Here, GnRH stimulates gonadotrope cells to secrete luteinizing hormone (LH) and follicle-stimulating hormone (FSH) in pulses. LH and FSH then act on target cells in the ovaries and testes, promoting gonadal growth and function. Gonadal steroids—estrogen (E2), progesterone (P), and testosterone (T)—exert feedback on GnRH secretion through neuroendocrine loops, either enhancing or inhibiting GnRH release. These hormones also enter the brain, where they appear to influence cognitive function. Two different populations of kisspeptin neurons, considered central pacemakers for GnRH neurons, surround GnRH neuronal somata and processes, and stimulate GnRH surges and pulsatile release, respectively. Neurons expressing neuronal nitric oxide synthase (nNOS) contribute to this rhythmic pattern by secreting nitric oxide (NO), a negative regulator of GnRH expression and release, a process also driven by kisspeptin. Ion channels and G protein-coupled receptors (GPCRs) expressed by GnRH neurons are also believed to play a role in regulating GnRH secretion. Neuroendocrine GnRH release is modulated by tanycytes, which control the access of GnRH terminals in the median eminence to the pericapillary space of pituitary portal capillaries, and astrocytes, which stimulate the electrical activity of GnRH neurons via prostaglandin E2 (PGE2). GnRH is also secreted into the cerebrospinal fluid (CSF), and GnRH neurons project to various GnRH-receptor-expressing brain areas, including the cortex and hippocampus, in keeping with its cognitive role. Interestingly, LH and FSH also reach the cortex and hippocampus and may also contribute to higher cognitive functions.

In mammals as in other vertebrate species studied, GnRH neurons do not originate in the brain but in the nasal region, specifically from the olfactory placode and neural crest, during embryonic development (2,30). These neurons then migrate towards the forebrain and hypothalamus, traveling along the fiber tracts of the olfactory and terminal nerves (31), in a process tightly regulated by spatially and temporally specific patterns of trophic factors, neurotransmitters, ion channels, adhesion molecules, attractive or repulsive guidance cues and their receptors (2). Secreted by glial cells, endothelial cells and neurons themselves, these factors not only provide the structural scaffold guiding GnRH neurons to their final destinations (2,32–35), they also play a critical role in regulating their autonomous migration, survival, connectivity and network formation, and function (36–39). Disruptions or mutations in these factors can lead to significant dysfunctions in the establishment or maturation of GnRH neurons, manifesting as impairments in reproductive capacity in humans, a condition known as congenital hypogonadotropic hypogonadism, or Kallmann syndrome when it is associated with the loss of the sense of smell (3). At the end of their migration, GnRH neurons occur as a loose continuum between the olfactory bulbs and the hypothalamus, with a particularly high concentration in the hypothalamic median preoptic area (MePO) (1,28,29). In primates, including humans, many GnRH neurons are also found in the tuberal region of the hypothalamus, close to the ME (40–42). These neurons, which possess a rare bipolar morphology only observed in adulthood in von Economo neurons in the brain of certain mammalian species (notably primates, cetaceans and elephants) (43), project one or both of their long “dendrons”, so named because they share both dendritic (e.g. reception of synaptic inputs) and axonal features (e.g. transmission of action potentials to neurosecretory terminals), to the ME, where these split up into short terminals that release their neurohormone into the fenestrated capillaries of the pituitary portal circulation (44–46) (Fig. 1). In addition to these neuroendocrine GnRH neurons, in the fetal human brain, around 8000 GnRH-immunoreactive neurons that may not participate in the regulation of the HPG axis are scattered across extrahypothalamic brain regions including the neocortex, hippocampus, thalamus and amygdala, although it is unclear whether these neurons persist or continue to express GnRH in adulthood, and what this putative non-neuroendocrine function might be (1). However, abundant GnRH-expressing neuronal processes have been identified in these extrahypothalamic regions (27,29,42), whose neurons express GnRH-R (26,27), supporting the existence of non-reproductive roles for GnRH. At least some of these processes appear to originate from the same neuroendocrine GnRH neurons that project to the ME (27), raising the intriguing possibility that regulatory processes that control GnRH release and notably its pulse/surge pattern might also influence its other roles.

In addition to GnRH itself, the modulation of LH and FSH by GnRH secretion is essential for its broader physiological roles, including its implication in both reproduction and cognition. The characteristics of GnRH/LH pulses, including their frequency and amplitude, are necessary and sufficient to induce ovulation and spermatogenesis (3). This intermittent pattern is essential to maintain the expression and signal transduction and prevent the desensitization of GnRH-R (47–49). GnRH/LH pulsatility varies across the different phases of the ovarian cycle in females (13,17,50), with a higher frequency during the follicular phase in humans and diestrus-proestrus in rodents, and a lower frequency during the luteal phase in humans and estrus in rodents.. In adult female rodents, LH pulses occur approximately every hour in diestrus-proestrus and every 180 min in estrus (17), while in humans, this pulsatile release occurs every 60 minutes during the follicular phase and every 90 minutes during the luteal phase (13). The same pituitary gonadotropes that secrete LH are also responsible for the pulsatile release of FSH, which is stored in distinct secretory granules (51,52). However, the regulation of FSH pulses by GnRH remains unclear, partly due to the difficulty in identifying FSH release episodes that can be clearly linked to GnRH release since most of the FSH present in the peripheral circulation is secreted in a constitutive manner soon after its synthesis. Additionally, GnRH-induced FSH release may differ from GnRH-induced LH release in terms of the frequency or amplitude of GnRH secretion required or the response time before secretion (see for review (53)), and the two gonadotropins are also differentially regulated by inhibins and activins (54). The pattern and levels of GnRH/LH release in women remain relatively stable until menopause, which typically occurs between the ages of 45 and 55, and is characterized by the complete depletion of ovarian follicles and cessation of reproductive capacity, accompanied by a progressive rise in FSH levels and the fluctuation and eventual decline of estrogen levels (55,56). During the perimenopausal period in women or immediately after oophorectomy/ovariectomy in both women and mice, the absence of estrogen-negative feedback from the ovaries leads to a consistently high GnRH/LH pulse frequency—around one pulse per hour in women (57–59), which is still within the range seen across the ovarian cycle in premenopausal women, and three pulses per hour in mice (17), while preovulatory LH surges in perimenopausal women become inconsistent due to hypothalamic-pituitary insensitivity to estrogen positive feedback (60). Several decades after menopause, there is a progressive and marked decrease in both the frequency and the amplitude of GnRH/LH pulses, possibly due to the functional exhaustion of hypothalamic GnRH neurons after this very long period of hyperactivity (59). While there is no defined timepoint at which reproductive function ceases in men, a progressive decline with age does occur, characterized by decreasing testosterone levels and increased LH pulse frequency (1 pulse every 90-120 min in young adults vs. 1 pulse every 60-90 min in >70 years old men), but an attenuation of LH pulse size (61), which appears to be due to diminished hypothalamic GnRH release without impairing GnRH action in healthy men (62).

### The neuroglial environment of GnRH neurons

Importantly, GnRH neurons are integrated within a complex regulatory neural network that determines their activity, including their rhythmic release of the hormone (6,63). This network includes two distinct populations of kisspeptin neurons, located in the anteroventral periventricular nucleus (AVPV) and in the arcuate nucleus of the hypothalamus (ARH, analogous to the infundibular nucleus in humans), respectively, as well as MePO neurons expressing neuronal nitric oxide synthase (nNos/*Nos1*), which together form an interconnected and synchronized neuronal ensemble. While the AVPV kisspeptin neuron population appears to act on GnRH neuronal somata in the MePO to stimulate GnRH production and its surge release in proestrous females, the ARH kisspeptin neuron population, which co-expresses neurokinin B (NKB) and dynorphin (the so-called “KNDy” neurons), sends stimulatory signals to GnRH dendrons projecting to the ME (reviewed in (6,64)). In parallel, kisspeptin-sensitive nNos neurons produce nitric oxide (NO), a rapidly diffusing gaseous messenger that acts as an intermittent brake on GnRH neuronal activity (reviewed in (65)). Along with intrinsic ultrashort feedback loops regulating ARH kisspeptin neuron activity (66), the alternation of these two opposing inputs onto GnRH neurons is thought to result in the regulation of pulsatile GnRH secretion in both males and females (67). In addition, the sudden release of a tonic NO brake, which keeps GnRH production and secretion under tight control during most of the estrous cycle in females, contributes to the preovulatory GnRH surge (68) (Fig 1.). Gonadal steroid feedback plays a crucial role in this complex and dynamic regulation of GnRH secretion, particularly through the biphasic effects of estrogen across the estrous cycle. During the early and mid-follicular phases or diestrus in mice, moderate levels of estrogen exert negative feedback on GnRH secretion, primarily through the suppression of kisspeptin release from KNDy neurons in the ARH (50,69,70), but also through estrogen-sensitive nNos neurons (71,72). In contrast, as estrogen levels peak just before ovulation, they exert positive feedback on GnRH production via enhanced AVPV kisspeptin neuron activity and the activity of MePO nNos neurons, which together are thought to synchronize the neuronal activity of neighboring GnRH neurons, triggering the preovulatory surge (6,64,65,73) (Fig. 1). While progesterone triggers surge levels of LH release during the early follicular phase and advances the midcycle LH surge in estradiol-implant-treated women (74,75), ovariectomized non-human primates (76), sheep (77) and rodents (78), during the luteal phase, progesterone represses GnRH pulse frequency in females (78–80). In sheep, the effects of progesterone are likely mediated by KNDy neurons (81), but this does not appear to be the case in mice (82). In males, the negative feedback regulation of pulsatile LH release by testosterone (83–85) also appears to involve KNDy neurons (86).

In addition to these regulatory neurons, gonadal-steroid-sensitive glial cells, specifically astrocytes and tanycytes in the hypothalamus, form an integral part of the cellular network that controls GnRH production and secretion (Fig. 1) (87). Astrocytes, including a subpopulation of glial progenitors that are recruited by GnRH neurons during the infantile period and accompany these neurons until adulthood (88,89), synthesize prostaglandin E2 (PGE2), which directly stimulates GnRH neuronal activity (90) and GnRH release, particularly during postnatal development and puberty onset (91–93). This regulation involves the activation of erbB receptors on astrocytes by circulating estrogens and growth factors, such as transforming growth factor α (TGFα) and the neuregulins (91–93). Moreover, astrocytes enhance their physical interactions with GnRH neurons and PGE2 synthesis through SynCAM1, an adhesion molecule whose expression is regulated by erbB4 signaling (88,94). These mechanisms— infantile astrocyte recruitment by GnRH neurons, prostaglandin and growth factor signaling, and astrocyte-to-GnRH neuron adhesion—are crucial for correct GnRH neuronal connectivity with afferent neurons, the precise regulation of hormonal secretion, and the timely onset of reproductive function (88,94). Intriguingly, astrocytic PGE2 signaling has also recently been shown to be modulated by kisspeptin through the direct activation of the canonical kisspeptin receptor Kiss1R in astrocytes to fine-tune the reproductive axis of mice (95). Tanycytes of the ME, specialized ependymoglial cells that express estrogen receptors (96), also play a key role in regulating GnRH neuron function (97). Their long processes guide and ensheath GnRH terminals and control their access to the pericapillary space of the pituitary portal circulation (98–101) by retracting in response to high circulating estrogen levels during proestrus as well as PGE2 released by endothelial cells, thus facilitating the surge release of GnRH necessary for ovulation (96,102,103). Complementing this process, Sema7A, whose expression in tanycytes is regulated by ovarian steroids, particularly progesterone, promotes GnRH terminal plasticity and retraction from the pericapillary space during diestrus, in a mechanism that involves β1-integrin signaling and is essential for the regulation of fertility (104). In parallel, Sema3A release by endothelial cells attracts GnRH neuronal processes towards the pericapillary space of the pituitary portal blood vessels to form direct neurovascular junctions (105). Tanycytes also respond to signals such as NO released by endothelial cells, which can induce rapid structural changes in their processes (103,106), while erbB receptors and TGFα signaling pathways in tanycytes further modulate these changes over longer timescales, contributing to the regulation of GnRH release (107). In addition, tanycytes, which also possess the ability to shuttle peripheral metabolic signals and hormones into the brain, allowing them to reach target neurons that control food intake and energy metabolism in the ARH and elsewhere (108), may be the missing link coupling the metabolic and reproductive axes, essential to optimize reproduction based on the availability of adequate energy reserves (97,109). For instance, leptin and ghrelin, two opposing metabolic hormones derived from peripheral fat stores and the gut, respectively, and transported into the hypothalamus by tanycytes (110–112), are integrated by ARH neurons to modulate appetite and energy homeostasis as well as the HPG axis (113,114). Interestingly, estrogen feedback through tanycytes has recently been found to be essential not only for the control of pulsatile GnRH/LH release but also for the appetite-suppressing effects of estrogens through the regulation of the activity of orexigenic NPY neurons in the ARH (96), further linking the two axes, with potential implications for cognitive function.

### Minipuberty, puberty and the establishment of GnRH neuron function

While neuroendocrine GnRH neurons reach their final positions before birth, they undergo a series of maturation processes throughout postnatal development that progressively alter their morphology, allow their integration into functional neural networks, enhance their biosynthetic abilities and refine their patterns of neurosecretion, changes that are crucial for the onset of puberty and sexual maturity (115). This postnatal maturational process involves the recruitment of newborn preoptic area astrocytes as “escorts” by GnRH neurons during infancy, a phenomenon that depends on prostaglandin D2 secreted by these neurons (88). This glial recruitment is indeed necessary for the correct connectivity of GnRH neurons with their glutamatergic afferents, and its disruption alters the first postnatal activation of GnRH neurons, a phenomenon termed “minipuberty”, and delays puberty, indicating that postnatal gliogenesis and communication between non-neuronal cells and GnRH neurons also shape the establishment of the neural networks controlling reproductive function (88,90,92). Postnatal sexual maturation has been categorized into four distinct stages, as previously outlined by Ojeda and colleagues in rodents (116): the neonatal period or the first week of extrauterine life, with the day of birth marked as postnatal day 0 (P0); the infantile period, from P8 until weaning, including notably minipuberty, when the first postnatal activation of GnRH neurons triggers a corresponding transient surge in GnRH production that results in early gonadal activation and lays the foundation for later puberty and fertility in both humans and other mammals (115,117); the juvenile period, during which the GnRH system is largely inactive in species with longer lifespans (3,118) but continues to mature in species with shorter lifespans such as rodents (119); and finally, the onset of puberty, characterized by the production of mature sperm in males and the first ovulation in females, marking the acquisition of reproductive capacity (119),(120).

Minipuberty, or the first transient and gonadal-steroid-independent activation of GnRH expression and the HPG axis during early postnatal development, is characterized by a marked surge in gonadotropins, in particular FSH, whose levels peak between 1 and 3 months in human infants and around 12 days in rodents (115,117). The minipubertal surge in gonadotropins drives the synthesis and release of gonadal steroids, including estrogens, which play a critical role in normal reproductive development (121–124). The termination of minipuberty remains less understood, but recent studies suggest that FSH-induced estrogen production activates a subset of estrogen receptor alpha (ERα)-expressing nNOS neurons (28,125), and the resulting release of NO reduces the amplitude and finally puts a stop to GnRH neuron activity through the activation of guanylate cyclase and subsequent membrane hyperpolarization (28,126). In mice lacking *Nos1*, FSH levels are abnormally elevated, accompanied by increased GnRH activity, delayed puberty, and disrupted estrous cyclicity, changes that can be reversed by inhaled NO or sildenafil treatment during the infantile period (28), suggesting that nNos activity is crucial for the negative feedback regulation of the HPG axis.

**Figure 2:**
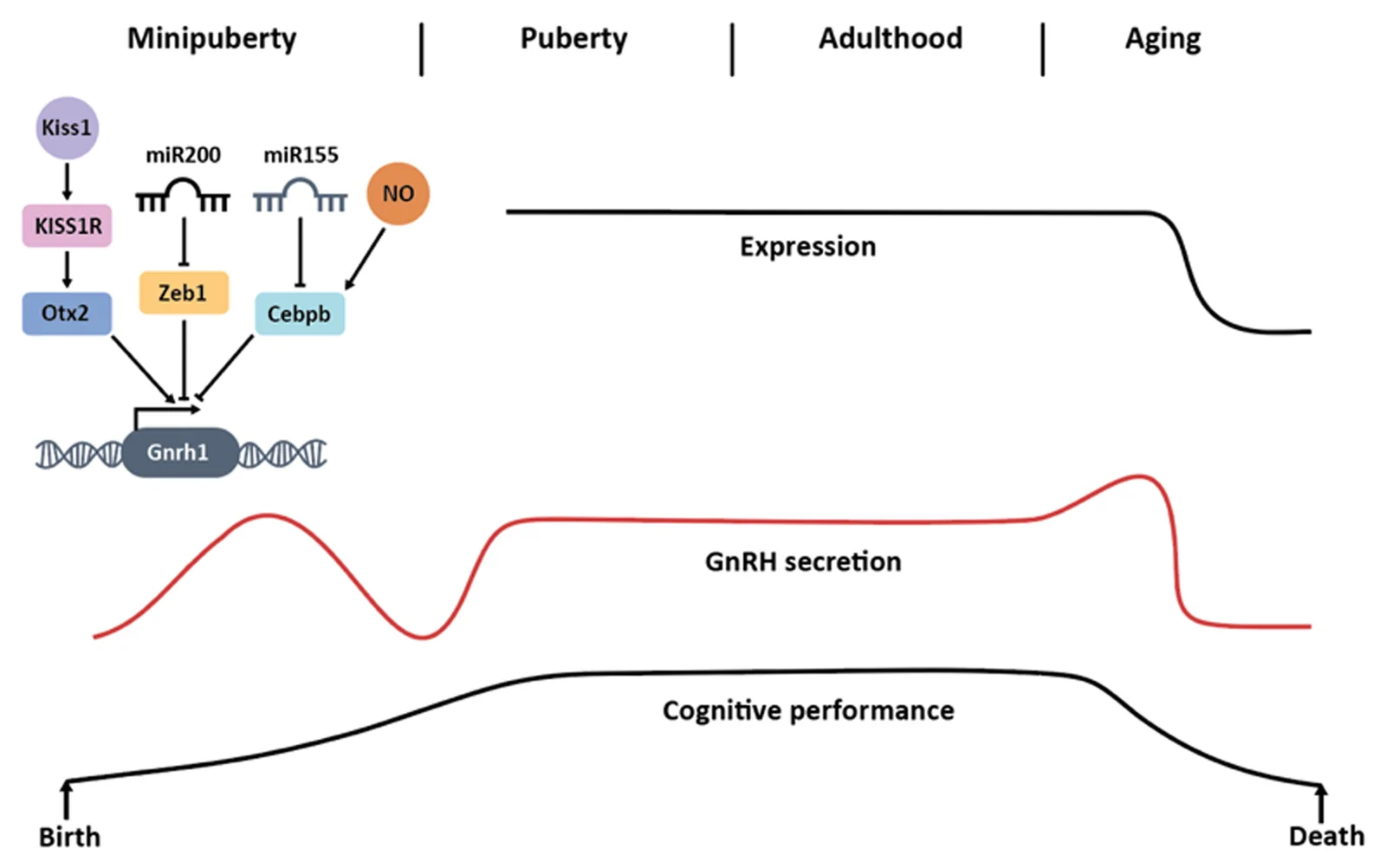
Regulation of GnRH expression and secretion, and associated cognitive effects across the lifespan. The activation of the GnRH promoter is finely regulated by a microRNA–transcription factor network, with miR-200/429 and miR-155 families essential for maintaining *Gnrh1* expression in GnRH neurons during minipuberty. These miRNAs modulate key transcriptional repressors (*Cebpb, Zeb1*) and the activator *Otx2*. Shifts in GnRH levels are mirrored by cognitive performance trends, which peak and stabilize by adulthood, followed by a gradual decline in later years.

At the molecular level, the minipubertal increase in the expression of GnRH depends on a critical regulatory switch consisting of the dramatic inversion in the expression of several microRNAs (miRNAs), which bind to and silence the translation of their target mRNAs, and transcription factors that act as either activators or repressors of the *Gnrh1* promoter (127). Two miRNA families and their feedback loops play a central role in this process: the miR-200/429 family, which is highly enriched in GnRH neurons and whose expression increases during this crucial period, and miR-155, which also shows elevated expression in hypothalamic GnRH neurons during minipuberty (127). Among the key transcription factors regulated by miR-200/429 and miR-155 are the *Gnrh1* promoter repressors genes *Zeb1* and *Cebpb* (which encodes the NO-mediated CAAT/enhancer-binding protein-β (C/EBPβ)), respectively (127,128). Additionally, these miRNAs also regulate transcriptional activators such as orthodenticle homeobox 2 (*Otx2*) (127), which promotes *Gnrh1* expression (129,130) and is known to define several developmental critical periods (131). Moreover, C/EBPβ and Zeb1 can also indirectly repress *Gnrh1* transcription by blocking kisspeptin signaling through Kiss1R, a receptor known to enhance *Gnrh1* transcription by promoting the nuclear translocation of Otx2 (127,132) (Fig. 2). This complex regulatory network ensures the precise control of GnRH production during minipuberty, and alterations in the expression of the component genes at minipuberty result in delayed puberty, hypogonadism and impaired fertility, highlighting the importance of this early phenomenon for sexual maturation and adult reproductive function.

In contrast to minipuberty, puberty, which represents the developmental phase in adolescence that culminates in reproductive maturity, is partly driven by the production of gonadal steroids (120,133) as well as a sustained increase in pulsatile GnRH release (134,135). During the peripubertal period, the emergence of a diurnal LH rhythm promotes ovarian maturation in females, and culminates in the full maturation of Graafian follicles, triggering the significant release of estrogens. These estrogens exert positive feedback on the HPG axis, triggering the first preovulatory surge of GnRH, LH, and FSH, ultimately leading to ovulation and the establishment of fertility. A key player in this process is the kisspeptin receptor Kiss1R (GPR54), a G-protein-coupled receptor essential for regulating reproductive function (Fig. 2) (136–138). Kisspeptin-Kiss1R signaling is crucial for maintaining normal pulsatile GnRH and LH secretion, as substantiated by studies using Kiss1R antagonists in various animal models (139). In primates, which display a long prepubertal quiescent period, KISS1R signaling also plays a role in triggering puberty by activating the pubertal resurgence of pulsatile GnRH release (140). Mutations in KISS1R and KISS1 in humans result in autosomal recessive idiopathic hypogonadotropic hypogonadism, underscoring the receptor’s importance for normal pubertal progression (136,137,141). Notably, kisspeptin immunoreactivity in neurons of the AVPV, which surround GnRH neurons, appears at the onset of puberty in rodents (142). Additionally, as mentioned in the previous paragraph, kisspeptin mediates the transcription of the *Gnrh1* gene through Otx2, a protein vital for GnRH neuronal development and the onset of puberty that remains active into adulthood, suggesting a role in maintaining GnRH expression (129,130,132). While the miRNA-transcription factor micronetwork switch flipped at minipuberty appears to be required for the lifelong expression of GnRH (27,127), additional epigenetic changes, such as DNA methylation and histone modifications, in other hypothalamic populations are involved in the control of puberty onset, including the pubertal rise in Kiss1 expression (143–148).

Both minipuberty and puberty are crucial for cognitive development as well as the establishment of reproductive function, with disruptions in their unfolding or timing leading to lasting effects that highlight the critical role of GnRH signaling in brain development and associated disorders, as further discussed below (115,149,150).

### The role of GnRH in brain development and cognition

Although traditionally linked to reproductive control, a number of recent findings suggest that GnRH may play a broader role in brain development and plasticity as well as cognitive processes. For instance, mice with a *Nos1* deficiency develop impairments in fertility, olfaction, hearing and cognitive performance, similar to traits observed in individuals with hypogonadotropic hypogonadism associated with *NOS1* mutations (28). Interestingly, supporting the role of NO in bringing minipuberty to an end by downregulating GnRH production, this *Nos1* deficiency is associated with exaggerated minipuberty (28,125). Restoring NO levels during the infantile period rescues not only sexual maturation but also cognitive and sensory performance in these mice, suggesting a broader role for minipuberty not only for the correct establishment of reproductive function but also the correct development and early programming of sensory, cognitive and behavioral networks in the brain (28). In this context, in the 2022 study on the role of GnRH in Down syndrome, in the Ts65Dn trisomic mouse model, the development of sensory and cognitive deficits mimicking the phenotype of Down syndrome patients (who are subfertile and in whom these deficits progressively appear or worsen from the minipubertal period) appears concomitant to the loss of GnRH expression that can be traced to a defective minipubertal GnRH switch (27). Indeed, human chromosome 21 and the orthologous trisomic region of mouse chromosome 16 both contain five miRNA genes, of which four, including miR-155, are highly expressed in GnRH neurons at minipuberty (127); miR-155 with its associated miRNAs and transcription factors constitutes one of the two main operators of the minipubertal switch, while the miR200 family is the other. The marked downregulation of postnatal *Gnrh1* expression in mice with trisomy of chromosome 16 is associated with the unexpected downregulation of the expression of the entire miR-200 family, which is not carried by chromosome 16 (or 21 in humans), in the preoptic region (27), indicative of the complex feedback loops connecting the networks (127). The selective rescue of miR-200 expression in the preoptic region using gene therapy reverses the phenotype, including the cognitive and behavioral performance of these mice, as does the restoration of physiologically relevant pulsatile GnRH levels using programmable osmotic minipumps (27). Interestingly, these positive effects appear to be independent of the stimulation of gonadal steroid production and action by pulsatile GnRH, since they are reproduced in orchidectomized mice (27), supporting a direct role for GnRH on brain regions involved in these functions. The relative contributions of such a direct role and an indirect effect through gonadal steroids remains to be determined experimentally.

Interestingly, in keeping with previous observations of GnRH-immunoreactive fibers in brain regions involved in higher cognitive functions in fetuses and newborns (1), both the abovementioned study and a follow-up study also reveal the presence of neuronal processes expressing GnRH and of neurons expressing its receptor, GnRH-R, in various cortical areas, the hippocampus, thalamus and amygdala, among other regions (27,29). Furthermore, the perturbation of genes involved in the minipubertal GnRH switch has been noted in other disorders. For example, NO and miR-155 expression is perturbed in the brain of ASD children (151,152), while ZEB1 expression is perturbed in the hypothalamus of patients with Prader-Willi syndrome (PWS) (153), a rare genetic disorder caused by the lack of expression of genes on the paternally inherited chromosome 15q11.2-q13 region, who present with cognitive disability and autistic symptoms as well as hypogonadotropic hypogonadism and eating disorders (154). While the hormonal profile at minipuberty seems normal in PWS infants (155,156), the progressive loss of hypothalamic GnRH neuron activity (156), a phenomenon also seen in the Ts65Dn Down syndrome model, evokes a possible GnRH switch deficit.

**Figure 3:**
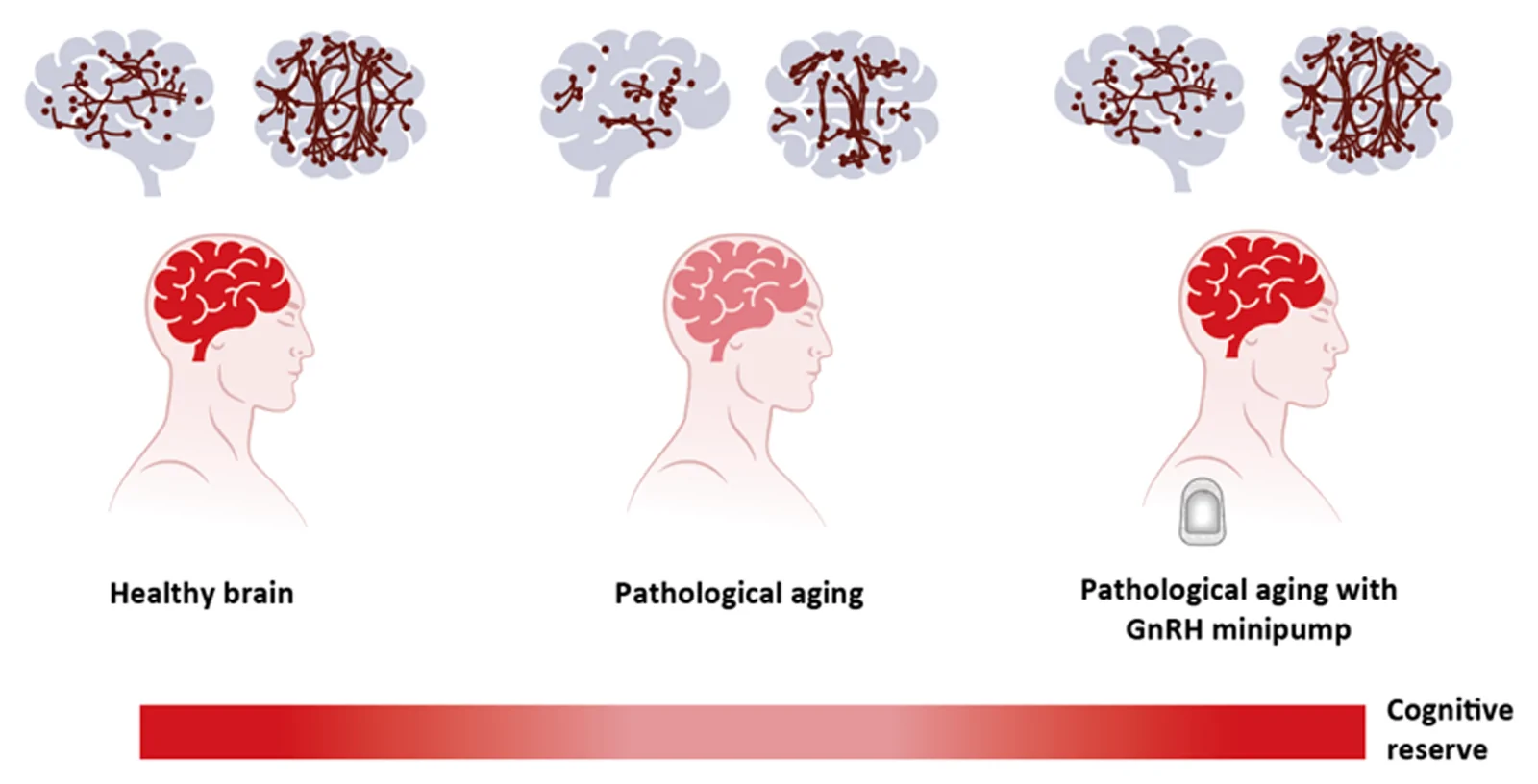
Pulsatile GnRH therapy enhances brain connectivity and cognition during aging. In pathological aging, brain connectivity and cognitive reserve typically decline. Pulsatile GnRH therapy is proposed to enhance connectivity between brain regions and improve cognitive function, as observed in Down syndrome patients (27).

The mechanisms by which GnRH might influence overall brain development and cognitive or behavioral functions include the modulation of gene expression and synaptic function and plasticity. In particular, while gonadal steroids and gonadotropins are suspected to contribute to white matter development during adolescence (157), recent research points to a more direct role for GnRH in maintaining brain myelination, even in adulthood. For instance, Down syndrome patients are also characterized by defective oligodendrocyte differentiation and hypomyelination (158). In Ts65Dn mice, correcting miR200 expression in the preoptic region restored *Gnrh1* promoter activity, corrected the expression of genes involved in oligodendrogenesis and myelination in the hippocampus, and improved hippocampal signal transmission (27). Furthermore, a pilot study of pulsatile GnRH administration using a programmable pump in seven young-adult volunteers with Down syndrome revealed not only cognitive improvements, but restored functional connectivity between several neocortical networks as detected by magnetic resonance imaging, possibly by promoting remyelination in some neuronal circuits (27). These improvements observed in brain structure and function with the use of pulsatile GnRH therapy, including rectified gene expression profiles, improved synaptic function and enhanced brain connectivity, may be linked to the mobilization of the “cognitive reserve”, a concept that refers to the brain’s ability to compensate for age-related or pathological changes through enhanced neural efficiency and adaptability (159) (Fig. 3). Interestingly, GnRH also appears to promote or sustain cell genesis, a phenomenon thought to be involved in functional plasticity, not only in the hypothalamus but also in the hippocampus in the adult mammalian brain (160). Together, these findings underscore GnRH’s potentially pivotal role in modulating neural connectivity and function throughout life, with important repercussions not only for reproduction but the correct establishment and maintenance of brain circuits involved in sensory and higher intellectual functions. The precise mechanisms underlying this role remain to be further explored both in animal models lacking GnRH-R in the brain as well as in patients with GnRH dysfunction of various etiologies.

Unexpectedly, the abovementioned 2022 study also uncovered the fact that, contrary to the beneficial effects of pulsatile GnRH administration on cognitive and sensory performance in mouse models of Down syndrome or Alzheimer’s disease (AD), which exhibit altered patterns of LH release (27), the continuous infusion of GnRH, shown by Knobil’s early studies to blunt pulsatile LH release (161), induces marked cognitive and sensory loss in young adult wild-type mice (27). This finding demonstrates the key role not only of GnRH but of its pattern of release in higher intellectual functions, and conversely, the pathological implications of alterations in this pulsatility. These novel insights help explain why GnRH agonists and antagonists, used to mimic continuous GnRH infusion and suppress the HPG axis in patients with steroid-hormone-responsive conditions such as prostate cancer, endometriosis or uterine fibroids, have been reported to exert adverse effects on cognitive function (162–167). In addition, prostate cancer treatment with these analogs of GnRH, known to bypass the blood-brain barrier (168), appears to increase the odds of developing dementia and AD (162,169,170).

GnRH agonist therapy is also widely employed for the management of central precocious puberty (120,171) and achieving puberty suppression in transgender children and adolescents (172). However, preclinical studies in sheep have revealed that the inhibition of puberty using GnRH agonists can result in sex-specific effects on brain development (173,174). These findings, along with clinical data reviewed elsewhere (149), highlight the pressing need for further investigations of the possible adverse effects of GnRH agonist treatment on brain development, learning and cognitive performance, and not just HPG axis function, in pediatric populations. This research is crucial to ensure the safety and advisability of these treatments in the long term, particularly given their increasing use in various clinical contexts.

### The HPG axis in physiological and pathological brain aging

Given the essential role that GnRH rhythmicity appears to play in cognitive and sensory processes (27) (see (149)for review), the dysfunction in GnRH signaling seen with age in men and women alike (59,61) may accelerate cognitive decline. Interestingly, the incidence of AD is higher in women than men; however, whether postmenopausal women are at increased risk of developing AD pathology or cognitive deficits is still a matter of debate (175–177). Age-related changes in GnRH neuron activity certainly appear to evolve with time in peri- and post-menopausal women, with an early and transient increase in the frequency of pulses followed by a later deceleration, as well as a gradual decrease in the amplitude of the pulses (59), possibly reflecting the “exhaustion” of GnRH neurons, as detailed above. While these frequencies largely overlap with the range seen in premenopausal women, it cannot be ruled out that their evolution plays a role in the observed cognitive decline or risk of developing AD, although these are not necessarily concomitant. Age at surgical menopause, where there is a premature loss of ovarian hormones following bilateral oophorectomy in women, has been shown to influence cognitive decline and AD pathology later in life (178), possibly due to accelerated aging (179,180). However, no association of cognitive performance with age at menopause was seen in women who underwent natural menopause (178). A 2024 meta-analysis concludes that women who experience menopause before the age of 40 years have a higher risk of dementia irrespective of the type of menopause (181). This apparent discrepancy might be explained by the hypothesis that age at natural menopause reflects the ‘biological’ age of the brain, which is based on GnRH neuronal function rather than chronological age. In other words, the brain’s aging process, as indicated by GnRH neuron function, may be more relevant to cognitive outcomes than the timing of menopause itself. Additionally, these changes in GnRH neuronal function with age likely also deregulate the expression of GnRH-R, which depends on normal GnRH pulsatility (47–49). Together with the decreasing amplitude of GnRH release, the desensitization or deregulation of GnRH-R in brain regions involved in cognitive or affective functions could further exacerbate age-related deficits. Further studies both of the evolution of GnRH pulsatility and amplitude with age in both men and women and the underlying mechanisms in preclinical models are required to fully understand these changes.

The early use of oral contraceptives or estrogen replacement therapy to prolong estrogen feedback from the perimenopausal period could potentially mitigate this issue (182). Hormone substitution therapy not only has beneficial effects with regard to adverse metabolic changes (183) but also clamps pulsatile GnRH/LH release at premenopausal levels (16). In mice, ovariectomy leads to reduced anterior-posterior connectivity as observed by functional magnetic resonance imaging (fMRI), reflecting a molecular phenotype indicative of accelerated aging (184). Additionally, ovariectomy has been seen to induce cognitive deficits by two months post-surgery, associated with hippocampal damage and specifically affecting long-term spatial memory as assessed by the Morris water maze (185), impairments that persist even seven months after surgery (186). Olfactory performance is also negatively affected following ovariectomy (187), and loss of olfactory perception is an early indicator of AD in human patients (188,189), as well as being associated with the absence or deficit of GnRH in both subjects with Kallmann syndrome and Down syndrome (3). It is worth noting that some postmenopausal women also demonstrate alterations in olfactory function (190). In men, low testosterone levels with age or androgen deprivation therapy with GnRH analogs (e.g. for prostate cancer) have similarly been associated with cognitive impairments and an increased risk of AD (162,169,170,191,192). However, trials with testosterone or estrogen replacement therapy in AD patients have shown inconsistent cognitive benefits, possibly because they do not compensate for the underlying stage-specific disruption in GnRH signaling (55,193–199). Preclinical studies suggest that, in menopaused women, elevated FSH levels, likely due to the age-related decrease in GnRH pulse frequency and increase in its pulse amplitude (200,201), could exacerbate AD-like Tau pathology and cognitive decline by inducing the expression of the *Gnrh1* promoter repressor C/EBPβ, a transcription factor that also promotes amyloid precursor protein cleavage (202,203). A 2024 study using the Thy1-ApoE4/Cebpb mouse model, which mimics sporadic AD without mutations in amyloid precursor protein or presenilin, highlights the significant role of C/EBPβ in driving several hallmarks of AD pathology in humans, including progressive cognitive decline, neurodegeneration and the formation of both amyloid plaques and neurofibrillary tangles, along with extensive neuroinflammation (204). In this context, GnRH has been suggested to have an anti-inflammatory effect, and its age-related loss may aggravate neuroinflammation and vice versa (160). Remarkably, the use of FSH-neutralizing antibodies in mouse models of AD reverses certain pathological changes (202,203), and another 2024 study has shown that the deletion of the FSH receptor prevents age-related memory decline in the 3xTg AD mouse model; intriguingly, this prevention was only partially abrogated in ovariectomized mice, suggesting the involvement of actors other than gonadal steroids (205). Intriguingly, individuals with Down syndrome, who are at very high risk of developing AD (206,207), have elevated levels of FSH despite having normal levels of testosterone and inhibin B, and pulsatile GnRH therapy normalizes circulating FSH levels in addition to boosting cognitive performance and resting state functional connectivity (27).

**Figure 4:**
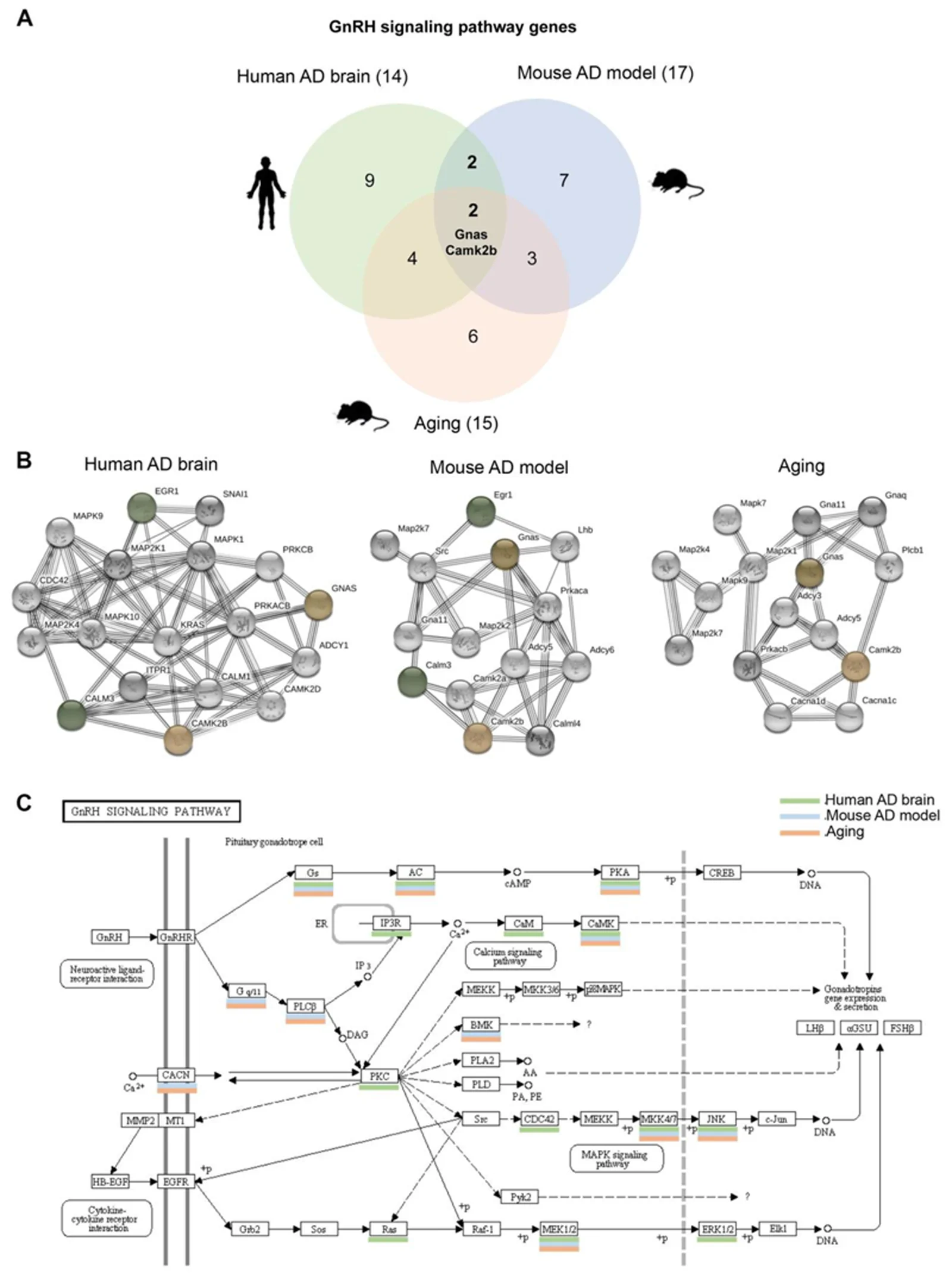
Transcriptional alterations of GnRH signaling pathway genes in Alzheimer’s disease and normal ageing. (A) Venn diagram showing the number of dysregulated genes associated with GnRH signaling in three datasets: downregulated genes in discrete cortical areas of post mortem brains of AD patients between low and high Braak neuropathological stages (208), as well as in the dorsal hippocampus of THY::TAU22 mice during learning (209); number of differentially-spliced GnRH signaling genes during normal aging in the mouse CA1 hippocampal region (210). (B) STRING analysis indicating the interaction between the GnRH-signaling-associated genes found in the three studies. Note that the Gnas and Camk2b genes were dysregulated in all three databases (orange) while Egr1 and Calm3 showed dysregulation in both the human AD brain and the mouse model of AD (green). (C) KEGG Pathway representing the significant number of genes involved in GnRH found to be downregulated in the human AD brain (green), in the dorsal hippocampus of the mouse AD model (blue) and/or differentially spliced during normal aging (orange).

Importantly, genes involved in GnRH signaling are among the most downregulated in the post mortem brain of AD patients (208) as well as in the *THY::TAU22* tauopathic mouse model of Alzheimer’s disease (Figure 4) (208–210). Similar changes, including *Cebpb* upregulation, have been observed in Ts65Dn mice (27), a model of Down syndrome that also mimics the AD-like pathology that these patients develop in their 40s (207), and, as mentioned above, Down syndrome patients, like AD patients, show elevated FSH levels (27). Recently, SARS-CoV-2 neuroinvasion has been shown to induce the death of GnRH neurons and a marked reduction in GnRH expression, which may underlie mental health impairments in long-COVID and heighten susceptibility to neurodevelopmental and neurodegenerative diseases, including AD or other forms of cognitive decline (211,212). It should also be remembered that GnRH neurons do not exist in a vacuum but as part of a complex neuroglial network, and that age-related or pathological deficits in other cells of this network may perturb GnRH secretion. For instance, tanycytes, which control the access of GnRH neuronal terminals to the pituitary portal circulation, and thus the rhythmicity of GnRH secretion (97), and play a role in transmitting estrogen feedback to hypothalamic circuits (96), are found to be specifically fragmented in the brain of AD patients (115), which may contribute to the HPG axis dysfunction observed in this disease. AD is also recognized as a multifaceted disorder, with metabolic and neuroendocrine alterations including changes in body weight, which often preceding cognitive decline and implicate hypothalamic dysfunction in the disease (213). Conversely, obesity, which could also be mediated by defective tanycytic function (110), is a risk factor for AD (214). The disruption of the tanycytic transport of metabolic hormones such as leptin and ghrelin into the brain, which both play an organizational role during brain development (215,216) and influence GnRH production and function (113,114) as well as acting on peripheral-hormone-responsive neurons in areas of the brain involved in cognition and behavior (217), may also contribute to perturbed GnRH signaling and cognitive decline in the aging or degenerating brain (211).

## Conclusion

GnRH secretion follows a distinct temporal course throughout life, beginning with a transient surge during minipuberty, followed by the establishment of its adult pattern at puberty and its function during the reproductive years, and eventual decline with age or after gonadectomy. The pulsatile nature of GnRH release, finetuned by the cellular and molecular environment surrounding and regulating GnRH neurons, is critical not only for reproductive function but also for cognitive processes. The emerging evidence for a key role for GnRH in myelination, synaptic transmission and cell genesis in the developing and adult brain also underscores its importance beyond the HPG axis. Both the frequency and amplitude of GnRH pulses are altered with age, particularly in postmenopausal women. This alteration may lead to the exhaustion of hypothalamic GnRH neurons, potentially contributing to cognitive decline. The therapeutic potential of restoring GnRH pulsatility, especially in neurodegenerative conditions such as Down syndrome and Alzheimer’s disease, creates new avenues for mobilizing the cognitive reserve and mitigating cognitive decline. Together with the putative adverse effects of altered minipuberty on overall brain development and function throughout life, these pinpoint the importance of further studying the establishment, maintenance and senescence of GnRH neurons and their network.
